# The Role of Algae Extract (*Ulva lactuca* and *Solieria chordalis*) in Fishmeal Substitution in Gibel Carp (*Carrassius auratus gibeilo*)

**DOI:** 10.3390/vetsci10080501

**Published:** 2023-08-03

**Authors:** Hualiang Liang, Hopeson Chisomo Kasiya, Dongyu Huang, Mingchun Ren, Lin Zhang, Heng Yin, Haifeng Mi

**Affiliations:** 1 Key Laboratory for Genetic Breeding of Aquatic Animals and Aquaculture Biology, Freshwater Fisheries Research Center (FFRC), Chinese Academy of Fishery Sciences (CAFS), Wuxi 214081, China; lianghualiang@ffrc.cn (H.L.); huangdongyu1995@163.com (D.H.); renmc@ffrc.cn (M.R.); zhangl@ffrc.cn (L.Z.); 2 Department of Aquatic Bio-Sciences, Graduate School of Agricultural and Life Sciences, University of Tokyo, Tokyo 13-8654, Japan; ntchindi@yahoo.com; 3 Tongwei Agricultural Development Co., Ltd., Key Laboratory of Nutrition and Healthy Culture of Aquatic Livestock and Poultry, Ministry of Agriculture and Rural Affairs, Healthy Aquaculture Key Laboratory of Sichuan Province, Chengdu 610093, China; yinh@tongwei.com

**Keywords:** Gibel carp (*Carrassius auratus gibeilo*), algae extract, fishmeal substitution, antioxidant capacity, immune capacity

## Abstract

**Simple Summary:**

The issue of fishmeal substitution has become increasingly important as the price of fishmeal has soared in recent years, driving up the cost of aquaculture feed and farming. However, fishmeal is one of the best protein sources, and problems such as growth inhibition and immune regulation disorders often occur after fishmeal substitution, which restricts a further reduction in the use of fishmeal. This study investigated the role of algal extract in fishmeal substitution and found that supplementation of algal extract when fishmeal reduction was effective in alleviating problems such as growth inhibition and reduced immunity. These results confirm that this algal extract can play an important role in fishmeal substitution, reduce feed costs, and provide a theoretical data basis for fishmeal substitution.

**Abstract:**

The function of algae extract (AE) in fishmeal (FM) substitution with plant proteins in the diets of Gibel carp (*Carrassius auratus gibeilo*) was investigated during a 56-day trial. Diets 1 and 2 contained 10% FM, Diets 3 and 4 contained 5% FM, and Diet 5 and 6 contained 0% FM. In contrast, Diets 2, 4, and 6 were supplemented with 0.2% AE. The results showed that FM reduction inhibited growth performance, while AE supplementation alleviated growth inhibition. FM reduction significantly decreased the crude protein levels of the whole body, while the contents of whole-body lipids were significantly decreased with AE supplementation. There were no significant changes in ALB, ALP, ALT, AST, TP, GLU, GLU, and TC in plasma. FM reduction with AE supplementation mitigated the decrease in antioxidant capacity by heightening the activity of antioxidant enzymes and related gene expressions, which mitigated the decrease in immune capacity by affecting the expression of inflammatory factors. In summary, AE supplementation could alleviate the negative effects of FM reduction in Gibel carp.

## 1. Introduction

Fishmeal (FM) is considered to be one of the highest-quality protein sources. It is widely used in aquaculture and high-grade animal feed. However, as one of the most expensive protein sources, FM’s price has increased in recent years, which has caused some problems for the aquaculture industry. For this reason, FM substitution has become a research hotspot. Various protein sources that could partially or completely replace FM have been evaluated in fish [1,2]. The animal protein source is an excellent protein source that can successfully achieve the partial or total replacement of FM. Animal protein ingredients could partially replace FM in the case of methionine and lysine supplement in Gibel carp (*Carassius auratus gibelio*) [3], and used poultry by-product meal could also reduce FM levels in Gibel carp [4] and rainbow trout (*Oncorhynchus mykiss*) [5]. Compared with animal protein sources, a plant protein source is cheaper and has better economy. Numerous studies have confirmed that plant protein sources could also replace partial FM, such as oriental river prawn (*Macrobrachium nipponense*) [6] and Atlantic salmon (*Salmo salar* L.) [7], whereas fish may not make good use of some plant protein sources due to anti-nutritional factors [8]. Some plant proteins considered for use as FM substitutes must be evaluated in terms of their nutrient content and possible harmful effects on fish health [9]. Certain plant protein sources contain antinutritional factors and toxic substances. Soybean meal has lectins and protease inhibitors [10]. Cottonseed meal contains free gossypol [11]. Rapeseed meal is a source of glucosinolate metabolites [12]. The foregoing substances may inhibit the growth and antioxidant and immune capacity of aquatic animals [13]. In recent years, researchers have explored various ways to attenuate the antinutritional properties of plant proteins destined as FM replacements. Enzymatic hydrolysis and fermentation helped augment utilization efficiency and diminish the toxicity of plant proteins [14,15]. Supplementation with certain functional substances also mitigated the harmful effects of plant protein sources [16,17].

Algae produce and store numerous active substances. Algae powder and the functional compounds therein have been administered to humans, animals, and plants and have proven beneficial. Algae powder and its derivatives and extract have been successfully integrated into pharmaceuticals, feed, functional additives, and fertilizers [18]. Algae extract (AE) regulates the growth and immunity of different aquaculture animals [19,20]; promotes overall health, antioxidant capacity, digestive enzyme production, and functions in fish and shrimp [21,22]; and elicited positive changes in the gastrointestinal microflora [23]. For example, *Ulva lactuca* has been found to be useful as a feed additive to combat heat stress in greenlip abalone (*Haliotis laevigata* Donovan) [24]. Furthermore, algae also play an important role in FM substitution, such as *Schizochytrium limacinum* in red drum (*Sciaenops ocellatus*) [25], *Solieria chordalis* and *Ulva lactuca* in olive flounder (*Paralichthys olivaceus*) [26], red algae (*Gracilaria arcuata*) in Nile tilapia (*Oreochromis niloticus*) [27], and *Ulva lactuca* in gilthead seabream (*Sparus aurata*) [28] and white-spotted snapper (*Lutjanus stellatus*) [29]. Algae meal and its extract are rich in bioactive substances [24] such as sulphated polysaccharides, vitamins, minerals, and peptides, which are beneficial to the growth, development, and immune regulation of aquatic animals. Hence, algae powder or its extract has high application value and potential in aquatic compound feed, whether as feed material or additives.

Gibel carp has excellent flesh quality and rapid growth performance, and it has become an important species of aquaculture in China [30]. Gibel carp as omnivorous fish requires relatively high dietary FM levels in their diet. Therefore, FM replacement is challenging, and some of the plant protein sources previously investigated inhibit the growth and the antioxidant and immune capacity of this fish species. Thus, the study aimed to investigate the role of AE in fishmeal substitution in Gibel carp (*Carrassius auratus gibeilo*).

## 2. Materials and Methods

### 2.1. Diet Preparation

The experimental design formula is shown in Table 1. Diets 1 and 2 contained 10% FM, Diets 3 and 4 contained 5% FM, and Diets 5 and 6 contained 0% FM, while Diets 2, 4, and 6 were added with 0.2% AE. All ingredients were crushed and sifted. Then, the raw material is then mixed with water and oil at the designed level and granulated on the feed machine. The feed is dried and stored in the refrigerator for later use.

### 2.2. Experimental Procedure

The study was conducted in a water re-circulation system at the base of Nanjing Agricultural University (Wuxi, China). Gibel carp were acclimatized to the culturing environment, and commercial feed (Tongwei Group, Wuxi, China) was administered for 15 d. There were three replicates per group. After the acclimatization period, healthy fish that were similar in size were selected and randomly assigned in triplicate to 1 of 24 tanks (450 L/tank), with 20 fish/tank. The fish were manually fed 2 mm long pellets at 07:30, 11:30, and 16:30 daily for 56 d (8 wks). The fish were fed at 3% of their body weight with respect to the amount of feed. An ambient natural photoperiod was used, and the levels of water temperature, pH, and dissolved oxygen were within the ranges of 24–26 °C, 7.3–7.8, and 5.6–6.5 mg/L, respectively.

### 2.3. Sample Collection

The sample collection method used was previously described [31]. Before experimental sampling, fish were fasted for 24 h, and then the fish from each tank was enumerated and weighed. Six experimental fish were selected per tank based on the principle of randomization. Of these, three were used for whole-fish composition analysis, and three were subjected to blood drawing and dissection under tricaine methanesulphonate (MS-222) anesthesia. Their intestinal and other tissues were sampled/harvested. Plasma was collected by centrifuging their blood at 3000 rpm (4 °C) for 10 min. All samples were kept at −80 °C until analysis.

### 2.4. Trial Analysis

The trial analysis method was conducted as previously described [31]. The levels of lipid, moisture, protein, and ash/gross energy were quantified using drying, Kjeldahl, Soxhlet, and combustion methods, respectively. The biochemical levels of plasma were also quantified using Mindray’s BS-400 automatic biochemical analyzer (Shenzhen, China). Antioxidant indices were measured as previously described [32]. The amino acid content in the experimental feed was quantified at Evonik Degussa Co. Ltd. (Shanghai, China) as previously described [33]. Table 2 lists the specific procedures, kits, and equipment used. The RNA of fish intestine was extracted using Takara’s RNAiso Plus Kit (Dalian, China) and qualitatively and quantitatively analyzed using Agilent Technologies’s NanoDropTM 2000 spectrophotometer (Santa Clara, CA, USA). Based on the description of the test, the target gene’s expression was evaluated using TaKaRa Bio Inc’s PrimeScriptTM RT Reagent Kit (Shiga, Japan). β-actin was the internal standard. To quantify the relative gene expression, the relative standard curve method was adopted similarly to a previous study [31]. Table 3 shows the used primers.

### 2.5. Statistical Analysis

SPSS 23 was used to evaluate data, and the results were presented as mean values and standard error (±SEM). One-way ANOVA followed by Tukey’s test was used to investigate the effects of FM reduction in AE supplementation and without AE supplementation groups, respectively, which is the significance of this study. Then, diet-related changes in various response parameters were investigated via two-way analysis of variance (ANOVA). *p* < 0.05 indicated significant differences.

## 3. Results

### 3.1. Growth Performance

The results of the growth assessment are shown in Table 4. FM reduction with and without AE supplementation did not significantly affect the survival rate (SR) (*p* > 0.05). FM reduction without AE supplementation significantly lowered the weight gain rate (WGR), specific growth rate (SGR), and final weight (FW) and raised the feed conversion ratio (FCR) (*p* < 0.05). In contrast, FM reduction with AE supplementation significantly alleviated growth inhibition compared to FM reduction alone (*p* < 0.05). The fish’s growth performance with respect to 5% FM administration with AE supplementation was similar to that of the fish with 10% FM administration. The fish administered 10% FM with AE supplementation exhibited the highest SGR and the lowest FCR of all treatment groups. There were no significant interactions between FM reduction with or without AE supplementation for all growth parameters (*p* > 0.05).

### 3.2. Body Composition

Table 5 shows the results of whole-body composition. Significantly lower crude protein and lipid levels of the whole body were observed in FM reduction without and with AE supplementation, respectively (*p* < 0.05). Significant interactions during FM reduction without AE and with AE supplementation were observed in the content of whole-body ash (*p* < 0.05).

### 3.3. Plasma Biochemistry

Table 6 shows the results of the plasma’s biochemistry. FM reduction with and without AE supplementation did not significantly influence the plasma’s contents: alanine aminotransferase (ALT), total cholesterol (TC), albumin (ALB), alkaline phosphatase (ALP), total protein (TP), aspartate aminotransferase (AST), glucose (GLU), and triglycerides (TG) (*p* > 0.05). There were no significant interactions among the foregoing parameters and FM reduction with and without AE supplementation (*p* > 0.05).

### 3.4. Intestinal Antioxidant Indexes

The antioxidant index measurements are listed in Table 7. FM reduction without AE supplementation significantly decreased the activity levels of manganese superoxide dismutase (Mn-SOD), catalase (CAT), total antioxidant capacity (T-AOC), glutathione peroxidase (GPx), total superoxide dismutase (T-SOD), and copper/zinc superoxide dismutase (Cu/Zn-SOD) and improved the malondialdehyde (MDA) content (*p* < 0.05). In contrast, FM reduction with AE supplementation increased the relative activity levels of the foregoing parameters. The MDA content was lower in response to FM reduction with AE supplementation than it was in response to FM reduction without AE supplementation. Significant interactions between FM reduction and AE supplementation were found in T-AOC and GPx (*p* < 0.05).

### 3.5. Relative Gene Expression

Table 8 presents the mRNA levels of genes. Significantly higher mRNA levels of interferon gamma (IFN-γ) were observed in the 5% FM group than in the 10% FM group (*p* < 0.05). Tumor necrosis factor alpha (TNF-α) and Mn-SOD mRNA levels were significantly higher and lower, respectively, in the 5% FM and 0% FM groups than in all other groups (*p* < 0.05). The 0% FM group presented a significantly lower GPx mRNA level than the 10% FM without AE supplementation group (*p* < 0.05). There were no significant differences between the FM reduction without AE supplementation groups in terms of their CAT and interleukin-10 (IL-10) mRNA levels (*p* > 0.05). The mRNA level of IFN-γ was significantly lower in the 10% FM with AE supplementation group than in the 5% FM group (*p* < 0.05). The Mn-SOD and CAT mRNA levels were significantly lower in the 0% FM without AE supplementation group than in the 10% FM without AE supplementation group (*p* < 0.05). No significant changes were found among the FM reduction with AE supplementation groups in terms of their TNF-α, GPx, and IL-10 mRNA levels (*p* > 0.05). Significant interactions were observed among the FM reduction with AE supplementation groups, the FM reduction without AE supplementation groups, and all foregoing parameters, except IL-10 and GPx (*p* < 0.05).

## 4. Discussion

In the present study, FM reduction without AE supplementation reduced growth performance; lowered FW, WGR, and SGR; and increased FCR. Proteins from plants contain antinutritional factors [34], which lowered the growth of fish. Here, the results of FW, WGR, SGR, and FCR of the 5% FM with AE supplementation group were similar to the 10% FM without AE supplementation group, indicating that AE supplementation can mitigate the negative effects of fish meal reduction in Gibel carp. Similarly to this study, *Solieria chordalis* and *Ulva lactuca*, as additives, could improve the growth performance of olive flounder in low-FM diets [26], while *Pyropia yezoensis* extract supplementation in low-FM diets improved growth and feed utilization compared with high-FM diets in these fish [35]. Furthermore, algae meal or its extract’s supplementation enhanced growth and feed utilization in Japanese flounder (*Paralichthys olivaceus*) [36] and olive flounder [37]. These results all supported our experimental results. A possible explanation is that algae meal and its extract are enriched in bioactive substances such as sulphated polysaccharides, vitamins, minerals, and peptides [24], which is beneficial to the digestibility and intestinal morphology of fish [26]. Furthermore, *Solieria chordalis* and *Ulva lactuca* supplementation could also improve digestive enzyme activity, such as amylase and lipase [26]. Similar studies have been found in white-spotted snappers fed with *Ulva lactuca* [29]. Hence, AE supplementation could also improve metabolic capability by increasing digestive enzyme activity. FM reduction without AE supplementation significantly decreased the whole-body crude protein content. Prior studies reported that *Ulva lactuca* and *Enteromorpha Linza* supplementation increased the crude protein content in ornamental goldfish (*Carassius auratus*), Nile tilapia, and rainbow trout [38,39,40,41]. *Chlorella*-supplemented diets significantly increased the crude protein content of muscle in fish [42], as FM reduction with AE supplementation significantly decreased the whole-body lipid content, indicating that AE might play an important role in lipid reduction. A previous report showed that fish fed a 5% *Ulva* meal diet presented significantly lower carcass lipid content than the control [40]. In fish research, blood biochemistry analyses help quantitatively evaluate fish health and nutritional metabolism. Here, no significant changes in plasma ALB, ALP, ALT, AST, TP, GLU, TG, or TC levels were found during FM reduction with and without AE supplementation, demonstrating that FM reduction with AE supplementation had limited effects on any of the foregoing blood biochemical indices.

ROS scavenging capacity depends on antioxidant enzyme activity, including T-AOC, CAT, SOD, and GPx, and it has a protective effect against oxidative damage [43,44,45]. Here, FM reduction without AE supplementation lowered the activity levels of T-AOC, GPx, T-SOD, and Mn-SOD. A low-FM diet reduced antioxidant enzyme activities in olive flounder [26]. Here, FM reduction without AE supplementation improved intestinal MDA levels. Earlier studies reported that FM reduction increased the MDA levels in juvenile grass carp (*Ctenopharyngodon idella*) [46], allogynogenetic Crucian Carp [47], and juvenile starry flounder (*Platichthys stellatus*) [48]. Certain plants may contain antinutritional factors, such as gossypol, proteinase inhibitors, phytosterols, and others [49,50]. Here, the activity level of enzymes was higher in the fish fed FM reduction with AE supplementation than in those fed with FM reduction without AE supplementation. Therefore, AE supplementation might have inhibited oxidative damage by upregulating the antioxidant enzymes that degrade ROS, such as superoxide ions and hydroxyl radicals [51]. Similarly to our study, *Solieria chordalis* and *Ulva lactuca* supplementation could also improve antioxidant enzyme activity [26], which supported our results. Here, AE supplementation might increase antioxidant enzyme activity, prevent oxidative damage, and protect against ROS in Gibel carp [52,53]. Similar results were also found in other fish. *Spirulina* supplementation in feed could also heighten intestinal antioxidant enzyme activity to prevent oxidative damage by inhibiting ROS formation [54]. Furthermore, *Gracilaria* spp. supplementation in feed could also enhance antioxidant capacity in European seabass (*Dicentrarchus labrax*) [55]. These studies support our experimental results. In addition to this, intestinal MDA levels were lower in the fish administered with FM reduction with AE supplementation than in those administered FM reduction without AE supplementation in the present study, which also supported the above results. Hence, AE supplementation might increase antioxidant enzymes to lower the body’s stress level. A possible explanation is that algae meal and its extract are rich in bioactive substances, such as sulphated polysaccharides [56,57], that regulate the antioxidant capacity and immunity in fish and shrimp [21,22,26].

The upregulation of antioxidant genes may improve the antioxidant capacity in fish [58]. Here, FM reduction without AE supplementation downregulated Mn-SOD and GPx, whereas FM reduction with AE supplementation counteracted these negative effects [59,60,61]. Thus, AE supplementation reversed the negative effects of FM reduction by improving antioxidant enzyme activity and, by extension, the antioxidant capacity. Cytokines regulate the inflammatory response in the body [62,63]. Here, FM reduction without AE supplementation upregulated TNF-α. Proinflammatory cytokines were upregulated in fish administered a low-FM diet [64,65,66]. In the present work, however, FM reduction with AE supplementation downregulated TNF-α. Hence, AE supplementation alleviated the post-FM reduction inflammatory response. In contrast, FM reduction with AE supplementation downregulated INF-γ possibly by attenuating the inflammatory response, triggering negative feedback and downregulating anti-inflammatory factors.

## 5. Conclusions

The present work showed that AE supplementation improved both the antioxidant and the immune capacity, thereby alleviating the adverse effects of FM reduction, such as growth inhibition, low antioxidant capacity, and immunosuppression in Gibel carp. The findings of this study lay theoretical and empirical foundations for future fish immunonutrition studies and provide insight into the regulatory mechanisms by which AE enhances the antioxidant and immune capacity in aquaculture fish.

## Figures and Tables

**Table 1:** Ingredient and nutrient composition of the experimental feed (% dry basis).

Kinds	Diet 1	Diet 2	Diet 3	Diet 4	Diet 5	Diet 6
Fish meal ^1^	10	10	5	5	0	0
Poultry meal ^1^	4	4	4	4	4	4
Soybean meal ^1^	16	16	20	20	23	23
Rapeseed meal ^1^	21	21	21	21	21	21
Cottonseed meal ^1^	10.5	10.5	14	14	17.5	17.5
Wheat bran ^1^	8	8	8	8	8	8
Wheat meal	14	14	12.7	12.7	11	11
Rice bran ^1^	8.13	7.93	5.53	5.33	4.69	4.49
Fish oil	2.39	2.39	3.3	3.3	3.86	3.86
Monocalcium phosphate	0.57	0.57	0.95	0.95	1.3	1.3
Mineral premix ^2^	2	2	2	2	2	2
Vitamin premix ^2^	0.8	0.8	0.8	0.8	0.8	0.8
35% ascorbyl-phosphate	0.1	0.1	0.1	0.1	0.1	0.1
60% Choline chloride	0.05	0.05	0.05	0.05	0.05	0.05
Mould inhibitor	0.05	0.05	0.05	0.05	0.05	0.05
Bentonite	2	2	2	2	2	2
L-Lysine ^3^	0.3	0.3	0.37	0.37	0.45	0.45
DL-Methionine ^3^	0.11	0.11	0.15	0.15	0.2	0.2
Algae extract ^4^	0	0.2	0	0.2	0	0.2
Analysed nutritional composition						
Crude protein (%)	33.05	33.07	33.09	33.13	33.03	33.05
Crude lipid (%)	6.37	6.31	6.36	6.32	6.33	6.35
Energy (MJ/kg)	17.48	17.5	17.52	17.51	17.53	17.52
Lys (%)	2.13	2.11	2.15	2.12	2.11	2.13
Met (%)	0.75	0.73	0.71	0.74	0.73	0.72

^1^ The protein levels of fish meal, poultry meal, soybean meal, rapeseed meal, and cottonseed meal were 60.1%, 65.5%, 45.1%, 42.0%, and 50.5%, and the lipid levels were 5.9%, 6.5%, 3.25%, 3.1%, and 2.8%, respectively, which were obtained from Wuxi Tongwei feedstuffs Co., Ltd. (Wuxi, China). ^2^ Wuxi Hanove animal health products Co., Ltd. (Wuxi, China) offered mineral premix and vitamin premix. ^3^ Evonik Industries AG (Hanau, Germany) offered DL-Methionine; Feeer Co., Ltd. (Shanghai, China) offered L-Lysine. ^4^ Olimx Group offered algae extract, a mature commercial product, named mFeed+. The main ingredients of the product include seaweed sulfide polysaccharide.

**Table 2:** Main methods and analysis equipment.

Item	Methods and Testing Equipment
Moisture	Drying method, oven, 105 °C
Protein	Kjeldahl method, Hanon K1100 auto Kjeldahl apparatus.
Lipid	Soxhlet method, Hanon SOX606 auto fat analyser
Ash	Combustion method, 550 °C for 5 h, XL-2A intelligent muffle furnace
Gross energy	Combustion method, IKA C6000 Oxygen bomb calorimeter
Alanine transaminase (ALT)	Mindray Bio Medical Co., Ltd.’s Assay kits, Mindray’s BS-400 automatic biochemical analyzer.
Total cholesterol (TC)	
Glucose (GLU)	
Alkaline phosphatase (ALP)	
Albumin (ALB)	
Triglyceride (TG)	
Aspartic transaminase (AST)	
Total protein (TP)	
Total antioxidant capacity (TAOC)	Jian Cheng Bioengineering Institute’s assay kits, Thermo Fisher Scientific microplate reader.
Superoxide dismutase (SOD)	
Catalase (CAT)	
Copper/zinc superoxide dismutase (Cu/Zn-SOD)	
Manganese superoxide dimutase (Mn-SOD)	
Malondialdehyde (MDA)	

**Table 3:** Primer sequences.

Primer	Forward 5′	Reverse 3′
Manganese superoxide dimutase (Mn-SOD)	AGCTGCACCACAGCAAGCAC	TCCTCCACCATTCGGTGACA
Glutathione peroxidase (GPx)	GAACGCCCACCCTCTGTTTG	CGATGTCATTCCGGTTCACG
Catalase (CAT)	CAGTGCTCCTGATACCCAGC	TTCTGACACAGACGCTCTCG
Interleukin-10 (IL-10)	GAGTCATCCTTTCTGCTCTGGTT	TTCATCGAGTAATGGTGCCAAGT
Interferon-γ (IFN-γ)	TCGCATGGAGAATGATAGTCTGG	GTCATCTTCCTTGATCGCCCATA
Tumor necrosis factor α (TNF-α)	TCATTCCTTACGACGGCATTT	CAGTCACGTCAGCCTTGCAG
β-actin	TCGTCCACCGCAAATGCTTCTA	CCGTCACCTTCACCGTTCCAGT

**Table 4:** Effects of FM supplementation with or without AE on growth performance of Gibel carp.

Indexes	Without AE			With AE			*p* Value		
	10FM	5FM	0FM	10FM	5FM	0FM	FM	AE	FM * AE
IW(g)	38.11 ± 0.09	37.99 ± 0.03	38.08 ± 0.07	38.10 ± 0.14	38.20 ± 0.13	37.99 ± 0.06	-	-	-
SR (%)	100 ± 0.00	100 ± 0.00	100 ± 0.00	100 ± 0.00	100 ± 0.00	100 ± 0.00	-	-	-
FW(g)	88.30 ± 1.27 ^c^	82.39 ± 1.08 ^b^	73.98 ± 0.71 ^a^	92.75 ± 0.63 ^C^	88.21 ± 0.62 ^B^	77.10 ± 1.08 ^A^	*p* < 0.001	*p* < 0.001	*p* = 0.373
FCR	2.11 ± 0.05 ^a^	2.28 ± 0.05 ^a^	2.82 ± 0.06 ^b^	2.01 ± 0.03 ^A^	2.11 ± 0.03 ^A^	2.59 ± 0.07 ^B^	*p* < 0.001	*p* < 0.005	*p* = 0.412
SGR (%/d)	1.22 ± 0.02 ^c^	1.12 ± 0.02 ^b^	0.96 ± 0.02 ^a^	1.29 ± 0.01 ^C^	1.21 ± 0.01 ^B^	1.03 ± 0.02 ^A^	*p* < 0.001	*p* < 0.001	*p* = 0.684
WGR (%/d)	131.68 ± 3.15 ^c^	116.87 ± 2.71 ^b^	94.28 ± 1.90 ^a^	143.29 ± 2.03 ^C^	130.93 ± 1.84 ^B^	102.96 ± 2.90 ^A^	*p* < 0.001	*p* < 0.001	*p* = 0.563

Note: Results are shown as mean values and standard error (±SEM). *p* < 0.05 indicates a significant difference. Different lowercase letters and uppercase letters in the same row indicate significant differences with respect to FM reduction without and with AE supplementation, respectively. The calculation formulas refer to previous studies [31]. * represents the interaction between FM and AE.

**Table 5:** Effects of FM supplementation with or without AE relative to the whole-body composition of Gibel carp.

Indexes	Without AE			With AE			*p* Value		
	10FM	5FM	0FM	10FM	5FM	0FM	FM	AE	FM * AE
Moisture (%)	78.73 ± 1.48	74.19 ± 2.52	78.81 ± 1.53	75.02 ± 1.15	78.70 ± 1.20	80.82 ± 2.17	*p* = 0.157	*p* = 0.523	*p* = 0.094
Protein (%)	14.05 ± 0.85 ^b^	14.25 ± 0.66 ^b^	10.47 ± 0.69 ^a^	11.21 ± 1.40	12.78 ± 0.87	12.06 ± 1.20	*p* = 0.202	*p* < 0.05	*p* = 0.697
Lipid (%)	6.38 ± 0.79	7.24 ± 0.69	6.14 ± 0.70	8.01 ± 0.44 ^B^	6.58 ± 0.68 ^AB^	5.19 ± 0.51 ^B^	*p* = 0.08	*p* = 0.99	*p* = 0.134
Ash (%)	2.71 ± 0.33	3.59 ± 0.25	3.16 ± 0.17	3.46 ± 0.09	3.14 ± 0.07	3.16 ± 0.27	*p* = 0.287	*p* = 0.966	*p* < 0.05

Note: Results are shown as mean values and standard error (±SEM). *p* < 0.05 indicated significant differences. Different lowercase letters and uppercase letters in the same row indicate significant differences relative to FM reduction without and with AE supplementation, respectively. * represents the interaction between FM and AE.

**Table 6:** Effects of FM supplementation with or without AE on the plasma biochemistry of Gibel carp.

Indexes	Without AE			With AE			*p* Value		
	10FM	5FM	0FM	10FM	5FM	0FM	FM	AE	FM * AE
ALB (g/L)	20.14 ± 2.80	19.59 ± 4.16	19.51 ± 2.98	20.56 ± 1.05	19.76 ± 2.64	19.92 ± 2.11	*p* > 0.05	*p* > 0.05	*p* > 0.05
ALP (mmol/L)	13.54 ± 1.62	15.59 ± 3.41	16.43 ± 1.99	15.46 ± 3.19	16.90 ± 3.20	17.69 ± 2.57	*p* > 0.05	*p* > 0.05	*p* > 0.05
ALT (U/L)	8.59 ± 1.43	8.94 ± 1.92	9.71 ± 1.41	11.29 ± 2.45	10.81 ± 2.5	9.51 ± 1.31	*p* > 0.05	*p* < 0.05	*p* > 0.05
AST (U/L)	268.44 ± 49.36	251.41 ± 25.66	218.50 ± 92.39	288.69 ± 68.69	276.44 ± 45.67	285.34 ± 64.95	*p* > 0.05	*p* > 0.05	*p* > 0.05
TP (g/L)	39.29 ± 5.10	38.85 ± 7.32	37.88 ± 5.59	40.86 ± 4.05	39.82 ± 4.38	39.65 ± 3.65	*p* > 0.05	*p* > 0.05	*p* > 0.05
GLU (mmol/L)	4.65 ± 1.96	4.89 ± 1.25	5.16 ± 1.75	4.56 ± 1.26	5.93 ± 1.84	5.56 ± 1.36	*p* > 0.05	*p* > 0.05	*p* > 0.05
TG (mmol/L)	2.89 ± 0.47	2.76 ± 0.54	2.67 ± 0.39	2.99 ± 0.38	2.61 ± 0.56	3.10 ± 0.84	*p* > 0.05	*p* > 0.05	*p* > 0.05
TC (mmol/L)	7.42 ± 0.95	7.29 ± 1.59	7.18 ± 1.26	7.69 ± 1.29	8.14 ± 1.69	7.57 ± 0.84	*p* > 0.05	*p* > 0.05	*p* > 0.05

Note: Results are shown as mean values and standard error (±SEM). *p* < 0.05 indicates significant differences. Different lowercase letters and uppercase letters in the same row indicate significant differences relative to FM reduction without and with AE supplementation, respectively. * represents the interaction between FM and AE.

**Table 7:** Effects of FM supplementation with or without AE on the intestinal antioxidant indexes of Gibel carp.

Indexes	Without AE			With AE			*p* Value		
	10FM	5FM	0FM	10FM	5FM	0FM	FM	AE	FM * AE
T-AOC (U/mg Protein)	7.98 ± 0.51 ^c^	5.13 ± 0.15 ^b^	2.05 ± 0.14 ^a^	9.27 ± 0.76 ^B^	7.02 ± 0.36 ^A^	5.65 ± 0.24 ^A^	*p* < 0.001	*p* < 0.001	*p* < 0.05
GPx (U/mg Protein)	361.08 ± 8.15 ^b^	260.16 ± 15.08 ^a^	253.59 ± 8.81 ^a^	387.75 ± 7.86 ^B^	355.17 ± 10.29 ^B^	293.67 ± 8.20 ^A^	*p* < 0.001	*p* < 0.001	*p* < 0.05
CAT (U/mg Protein)	58.86 ± 2.82	72.03 ± 4.58	62.28 ± 3.65	67.61 ± 3.13	87.50 ± 2.53	72.92 ± 3.03	*p* < 0.001	*p* < 0.001	*p* = 0.558
T-SOD (U/mg Protein)	70.90 ± 0.99 ^b^	66.87 ± 1.67 ^ab^	64.54 ± 1.05 ^a^	78.49 ± 0.64 ^B^	74.64 ± 1.69 ^AB^	72.34 ± 1.32 ^A^	*p* < 0.001	*p* < 0.001	*p* = 0.997
Cu/Zn-SOD (U/mg Protein)	7.13 ± 0.15	7.63 ± 0.12	7.83 ± 0.28	9.97 ± 0.35	10.36 ± 1.10	9.29 ± 0.77	*p* = 0.678	*p* < 0.001	*p* = 0.404
Mn-SOD (U/mg Protein)	63.76 ± 0.98 ^b^	59.24 ± 1.60 ^ab^	56.71 ± 1.06 ^a^	68.52 ± 0.97 ^B^	64.27 ± 2.35 ^AB^	63.05 ± 0.74 ^A^	*p* < 0.05	*p* < 0.001	*p* = 0.198
MDA (nmol/mg Protein)	12.39 ± 0.82 ^a^	14.41 ± 0.63 ^a^	16.65 ± 0.55 ^b^	9.28 ± 0.49	11.56 ± 0.70	11.43 ± 0.74	*p* < 0.001	*p* < 0.001	*p* = 0.342

Note: Results are shown as mean values and standard error (±SEM). *p* < 0.05 indicated a significant difference. Different lowercase letters and uppercase letters in the same row indicate significant differences during FM reduction without and with AE supplementation, respectively. * represents the interaction between FM and AE.

**Table 8:** Effects of FM supplementation with or without AE relative to the intestinal gene expression of Gibel carp.

Indexes	Without AE			With AE			*p* Value		
	10FM	5FM	0FM	10FM	5FM	0FM	FM	AE	FM * AE
TNF-α	1.13 ± 0.03 ^a^	1.18 ± 0.03 ^a^	1.79 ± 0.10 ^b^	1.02 ± 0.05	1.13 ± 0.04	1.06 ± 0.05	*p* < 0.001	*p* < 0.001	*p* < 0.001
IL-10	0.43 ± 0.02	0.40 ± 0.02	0.36 ± 0.03	0.33 ± 0.02	0.32 ± 0.04	0.33 ± 0.02	*p* = 0.411	*p* < 0.05	*p* = 0.432
IFN-γ	1.39 ± 0.14 ^a^	3.88 ± 0.12 ^b^	1.74 ± 0.12 ^a^	1.16 ± 0.05 ^A^	2.72 ± 0.10 ^B^	1.20 ± 0.07 ^A^	*p* < 0.001	*p* < 0.001	*p* < 0.05
Mn-SOD	0.40 ± 0.01 ^b^	0.20 ± 0.01 ^a^	0.18 ± 0.01 ^a^	0.36 ± 0.02 ^B^	0.35 ± 0.01 ^B^	0.15 ± 0.02 ^A^	*p* < 0.001	*p* = 0.077	*p* < 0.001
GPx	0.33 ± 0.02 ^b^	0.28 ± 0.01 ^ab^	0.23 ± 0.02 ^a^	0.42 ± 0.02	0.35 ± 0.03	0.34 ± 0.01	*p* < 0.001	*p* < 0.001	*p* = 0.061
CAT	0.79 ± 0.04	0.67 ± 0.02	0.72 ± 0.03	0.95 ± 0.06 ^B^	0.93 ± 0.05 ^AB^	0.72 ± 0.05 ^A^	*p* < 0.05	*p* < 0.05	*p* < 0.05

Note: Results are shown as mean values and standard error (±SEM). *p* < 0.05 indicated significant differences. Different lowercase letters and uppercase letters in the same row indicate significant differences relative to FM reduction without and with AE supplementation, respectively. * srepresents the interaction between FM and AE.

## Data Availability

The authors confirm that the data supporting the findings of this study are available within the manuscript and tables.
